# A Bayesian network model to predict the role of hospital noise, annoyance, and sensitivity in quality of patient care

**DOI:** 10.1186/s12912-022-00948-5

**Published:** 2022-09-01

**Authors:** Milad Abbasi, Saied Yazdanirad, Mojtaba Zokaei, Mohsen Falahati, Nazila Eyvazzadeh

**Affiliations:** 1 Faculty of Paramedicine, AJA University of Medical Sciences, Tehran, Iran; 2 Occupational Health and Safety Engineering, Social Determinants of Health Research Center, Saveh University of Medical Sciences, Saveh, Iran; 3 School of Health, Shahrekord University of Medical Sciences, Shahrekord, Iran; 4 Modeling in Health Research Center, Shahrekord University of Medical Sciences, Shahrekord, Iran; 5 Radiation Sciences Research Center, Faculty of Paramedicine, AJA University of Medical Sciences, Tehran, Iran

**Keywords:** Bayesian network, Noise exposure, Annoyance, Sensitivity, Quality of patient care, Nurse

## Abstract

**Background:**

Hospital noise can adversely impact nurses’ health, their cognitive function and emotion and in turn, influence the quality of patient care and patient safety. Thus, the aim of this study was to predict the contributing roles of exposure to hospital noise, staff noise-sensitivity and annoyance, on the quality of patient care.

**Methods:**

This descriptive and cross-sectional study was carried out among nurses in an Iranian hospital. To determine nurses’ noise exposure level, the noise was measured in 1510 locations across the hospital in accordance with ISO 9612 standards using KIMO DB 300/2 sound level meter and analyzer. An online survey was used to collect nurses’ individual data. Study questionnaires included demographics, Weinstein noise sensitivity scale, noise annoyance scale, and quality of patient care scale. Finally, to analyze the data, Bayesian Networks (BNs), as probabilistic and graphical models, were used.

**Results:**

For the high noise exposure state, high noise sensitivity, and high annoyance, with the probability of 100%, the probability of delivering a desirable quality of patient care decreased by 21, 14, and 23%, respectively. Moreover, at the concurrently high noise exposure and high noise sensitivity with the probability of 100%, the desirable quality of patient care decreased by 26%. The Bayesian most influence value was related to the association of noise exposure and annoyance (0.636). Moreover, annoyance had the highest association with the physical aspect of quality of care (0.400) and sensitivity had the greatest association with the communication aspect (0.283).

**Conclusion:**

Annoyance induced from environmental noise and personal sensitivity affected the quality of patient care adversely. Moreover, noise and sensitivity had a separate direct adverse effect upon the quality of patient care, and their co-occurrence reduced the potential for delivering quality patient care.

## Background

Noise, as unwanted and disturbing sound, is considered an environmental stressor for people’s health. In recent years, many studies have reported that the noise pollution level in hospitals is rising and becoming an adverse factor for patient and staff health [1–3]. Regarding this, Florence Nightingale expressed that “unnecessary noise is the most cruel absence of care which can be inflicted on the sick or well” [4]. Various sources of sound, including that produced by hospital equipment, people, facility infrastructure, and local traffic noise in high density areas contribute to rising noise levels for staff and patients – often beyond recommended guideline limits [5]. Based on the WHO guideline, the noise levels in hospital wards should not exceed 35 dB (dBA) during daytime and 30 dB during nighttime. Moreover, it is recommended that the allowable peak noise level be no more than 40 dB at any time [6]. Increased noise levels beyond these limits can disturb nurses’ performance and productivity. Higher background noise levels can disturb signal-to-noise ratio and in turn, intelligible communication. Ineffective and disrupted communication is one of the important factors negatively impacting nurses’ performance and productivity [6–8]. Moreover, noise level exceedance above these limits may have adverse health effects on the patients and staff. Morrison et al. in a 2003 review concluded that hospital noise can trigger annoyance, stress, tachycardia, and higher heart rates in nurses [9]. Another study concerning the effect of hospital noise on nurses’ health reported that 14.49% of nursing professionals complained about tinnitus, 34.78% about low concentration, 45.63% about auditory irritation, and 44.20% about sleep disturbances [5, 10]. Moreover, high noise level has a significant and negative effect on quality of patient care, nurses’ mental workload and anxiety levels [11, 12]. Cognitive functions such as memory, attention, concentration and problem solving are also influenced by exposure to noises with various characteristics such as frequency, content, and intensity [13, 14]. These adverse health effects induced by chronic exposure to noise levels above recommended guidelines have a synergistic effect, disturbing nurses’ performance and quality of patient care. Noise can influence the quality of patient care directly and also indirectly through the adverse health outcomes.

In addition to noise exposure, individual sensitivity to noise can intensify the effects and decrease performance. Abbasi et al. showed that noise sensitivity significantly increased noise annoyance and job stress and also decreased job satisfaction [15]. This is because noise-sensitive people are vulnerable to noise and other environmental stressors [16]. Therefore, hospital noise can threaten nurses’ health, their cognitive function and emotion, and in turn, influence the quality of patient care and patient safety. Despite the importance of the effect of noise on the quality of patient care, there is a paucity of a strong literature base on this issue. Thus, the aim of this study was to predict the contributing roles of exposure to hospital noise, staff noise sensitivity and annoyance, on the quality of patient care.

## Methods

### Study population

This descriptive and cross-sectional study was carried out on 209 nurses in 2021 in one hospital in Tehran, Iran. Noise exposure data were gathered in a four-month period from April 10, 2021, to August 10, 2021. The study process began following preliminary measures such as obtaining permission from the research ethics committee of Aja University of Medical Sciences (IR.AJAUMS.REC1400.060) and also getting permission from the hospital management. Questionnaires were distributed to staff nurses. These questionnaires included demographic information, noise sensitivity, noise annoyance, and quality of patient care. Since this study was conducted during the COVID-19 pandemic, in order to comply with preventive measures, the questionnaires were offered to the nurses based on their preference as an online link or printed hard copy. For those who opted for digital access, the questionnaire link was sent by hospital management to all nurses as they had access to the nurses’ contact information. In both delivery formats, the goals and way of participating in the study were explained and nurses were invited to complete the questionnaires voluntarily. Finally, nurses with less than one year of work experience, incomplete surveys, nurses with dual employment, or those with a restrictive disease were excluded from the study. The online questionnaires were prepared and sent to all nurses employed in the studied hospital. To encourage increased participation, also printed hard copy questionnaires were distributed among voluntary nurses who did not complete the online questionnaire.

### Questionnaires

Nurses completed the following self-report questionnaires.

### Demographic questionnaire

The demographic questionnaire collected information such as age, experience, gender, marital status, education, and working department.

### Weinstein noise sensitivity scale

To determine noise sensitivity, Weinstein noise sensitivity scale was used. This scale includes items related to attitude and affective reactions to noise in the various situations. It consists of 21 items with a 6-point Likert scale ranging from “agree strongly” to “disagree strongly”. The total score ranging from 0 to 105 can be categorized into three groups of low sensitivity (scores less than 25th percentile), moderate sensitivity (scores ranging from 25th percentile to 75th percentile), and high sensitivity (scores more than 75th percentile). Alimohammadi et al. investigated and confirmed the reliability and validity of the Persian translation of this scale [17].

### Noise annoyance scale

Moreover, a 100-point visual analogue scale with two verbally labelled poles ranging from “not at all annoying” to “extremely annoying” was used to assess nurses’ noise annoyance [18]. In this single item measure, nurses rated their perceived annoyance due to hospital noise on a 100-point graphical rating scale. The total score of noise annoyance was stratified as low (scores less than 25th percentile), moderate (scores ranging from 25th percentile to 75th percentile), and high annoyance (scores more than 75th percentile).

### Quality patient care scale

Quality patient care scale [19] is a standard tool consisting of 65 questions in three dimensions including psychosocial (28 items), communicational (13 items) and physical (24 items). The items were answered based on a 4-point Likert-type ranging from 1 to 4 as rarely (1), sometimes (2), often (3), and always (4). The total score of quality of patient care ranged from 65 to 260 and the higher scores indicated higher quality of patient care. Scores less than 130 were considered as undesirable, 130–195 as partly undesirable, and 196–260 as desirable. Regarding the psychosocial aspect, scores less than 56 were considered as undesirable, 56–84 as partly undesirable, and 85–112 as desirable. Regarding the communicational aspect, scores less than 26 were considered as undesirable, 26–39 as partly undesirable, and 40–52 as desirable. Moreover, the physical aspect score was categorized as undesirable (scores less than 48), partly undesirable (scores from 48 to 72), and desirable (scores from 73 to 96). The reliability of this questionnaire was checked, with a Cronbach’s alpha coefficient of 0.94 for this study.

### Noise measurement procedure

To plot the noise map, measurement points (1510 points five meters apart) were determined on the hospital building map and the noise was measured at all points across different departments. Measurement was performed in accordance with ISO 9612 using KIMO DB 300/2 sound level meter and analyzer. Beginning April 2021, a one-minute equivalent continuous sound pressure level (Leq,_1 min_) was measured at each point ten times over the course of the day: every two hours from 08:00 to 22:00 to cover all noise level changes during the whole day, and twice during the nighttime shift from 00:00 to 08:00 as representative of the noise level in this shift. This process continued until sound measurements from all 1510 points were obtained. Therefore, to determine noise level at each point, measurements were collected ten times for each point, resulting in a total of 250 hours of noise measurement. The logarithmic mean for each point was calculated and a noise map was plotted.

To determine nurses’ noise exposure, nurses who participated in the study were asked to specify their workstations and the duration of their work at that point. Using these data and referring to the ISO 9612, the equivalent continuous sound pressure level (LAeq) was calculated by the LAeq equation. Finally, the daily personal noise exposure level (LEP,d) was calculated for each nurse. Nurses’ noise exposures with values less than 50 dBA, 50 to 60 dBA, and higher than 60 dBA were considered as low exposure, moderate exposure, and high exposure, respectively.

### Statistical analysis

The statistical tests were carried out using SPSS software version 24 [20]. Descriptive statistics were calculated. Then the expectation-maximization method was applied to calculate and replace the missing values.

Bayesian Networks (BNs), as probabilistic and graphical models, were introduced by Pearl [21]. In this study, GeNIe academic software version 2.3 was used to analyze Bayesian network. After drawing the BN graphical structure, a Conditional Probability Table (CPT) was obtained by the model with the Expectation-Maximization algorithm [22]. Then, delta p sensitivity analysis was applied to rank the parameters [23]. Finally,10-fold cross-validation analysis was exploited to examine the model validity. The dataset was randomly divided into ten equal folds, nine folds (9 subsamples) were applied to train the Bayesian network model, and the remaining fold (1 subsample) was used to validate the model. A sensitivity analysis also was conducted to examine the effects of the variables [24].

## Results

This cross-sectional study was conducted in 2021 among nurses of Imam Reza Hospital affiliated with the Aja University of Medical Sciences. Of the total number of nurses employed, 209 nurses fully completed the questionnaires. The mean ± standard deviation (SD) of the age and experience were 36.10 ± 7.43 and 11.33 ± 6.87, respectively. Table 1 presents the demographic characteristics of nurses.

**Table 1:** Demographic characteristics of the participants

Variable		Frequency	Relative frequency
Age (years)	Less than 30 years	96	45.90%
	30 to 50 years	71	34.0%
	More than 50 years	42	20.10%
Job experience (years)	Less than 10 years	98	46.90%
	10 to 20 years	92	44.00%
	More than 20 years	19	9.10%
Gender	Male	88	42.10%
	Female	121	57.90%
Marital status	Married	107	51.20%
	Single	102	48.80%
Education level	Bachelor degree	152	72.70%
	Higher than Bachelor degree	57	27.30%
Department	ICU	49	23.40%
	Emergency	24	11.50%
	Surgery	39	18.70%
	Internal medicine	29	13.90%
	Blood transfusion	31	14.80%
	Others	37	17.70%

The mean ± SD of the noise exposure, noise annoyance and noise sensitivity for all nurses were 54.72 ± 4.40, 57.51 ± 21.93, and 59.02 ± 18.80 respectively. Moreover, the mean ± SD of total quality of patient care, psychosocial, communicational and physical aspects were 187.84 ± 32.59, 76.45 ± 15.22, 39.35 ± 7.35 and 71.60 ± 15.54 respectively. These variables were categorized and the mean ± SD per each group is presented in Table 2.

**Table 2:** Mean ± standard deviation of the studied variables

Variable		Frequency	Percent	Mean	SD
Noise exposure	Low	47	22.5%	48.80	1.84
	Moderate	126	60.3%	54.92	1.69
	High	36	17.2%	61.75	1.53
Noise sensitivity	Low	59	28.2%	34.98	7.62
	Moderate	93	44.5%	60.08	6.10
	High	57	27.3%	82.17	5.81
Noise annoyance	Low	61	29.2%	32.95	8.23
	Moderate	104	49.8%	58.75	7.96
	High	44	21.1%	88.63	15.18
Quality of patient care (total score)	Undesirable	19	9.1%	119.52	5.31
	Partly desirable	97	46.4%	175.83	18.78
	Desirable	93	44.5%	214.32	14.29
Psychosocial aspect	Undesirable	24	11.5%	47.16	8.20
	Partly desirable	116	55.5%	73.16	6.81
	Desirable	69	33.3%	92.18	5.94
Communicational aspect	Undesirable	21	10.0%	24.95	1.96
	Partly desirable	101	48.3%	36.37	3.32
	Desirable	87	41.6%	46.29	2.66
Physical aspect	Undesirable	30	14.4%	43.46	5.17
	Partly desirable	74	35.4%	65.41	5.49
	Desirable	105	50.2%	84.00	6.62

The results indicate that the highest frequency in each quality of patient care category was: partly desirable quality of patient care total, partly desirable psychosocial aspect, partly desirable communicational aspect and desirable physical aspect. In term of physical aspects of care, 50.2, 35.4 and 14.4% of nurses provided desirable, partly desirable and undesirable levels of care, respectively. Table 3 also represents the Conditional Probability Table (CPT) for quality of patient care, which describes the coefficients among the variables.

**Table 3:** The conditional probability table (CPT) for overall quality of patient care

Noise exposure	Noise sensitivity	Noise annoyance	Undesirable	Partly undesirable	Desirable
Low	Low	Low	0.037	0.333	0.630
		Moderate	0.333	0.333	0.334
		High	0.333	0.333	0.334
	Moderate	Low	0.167	0.083	0.750
		Moderate	0.500	0.333	0.167
		High	0.333	0.333	0.334
	High	Low	1.000	0.000	0.000
		Moderate	0.000	1.000	0.000
		High	0.333	0.333	0.334
Moderate	Low	Low	0.000	0.231	0.769
		Moderate	0.118	0.353	0.529
		High	0.333	0.333	0.334
	Moderate	Low	0.000	0.200	0.800
		Moderate	0.038	0.481	0.481
		High	0.333	0.333	0.334
	High	Low	0.500	0.500	0.000
		Moderate	0.136	0.409	0.455
		High	0.000	0.833	0.167
High	Low	Low	0.333	0.333	0.334
		Moderate	0.000	1.000	0.000
		High	0.333	0.333	0.334
	Moderate	Low	0.000	1.000	0.000
		Moderate	0.000	1.000	0.000
		High	0.273	0.455	0.273
	High	Low	0.333	0.333	0.334
		Moderate	0.000	0.000	1.000
		High	0.111	0.889	0.000

**Fig. 1:**
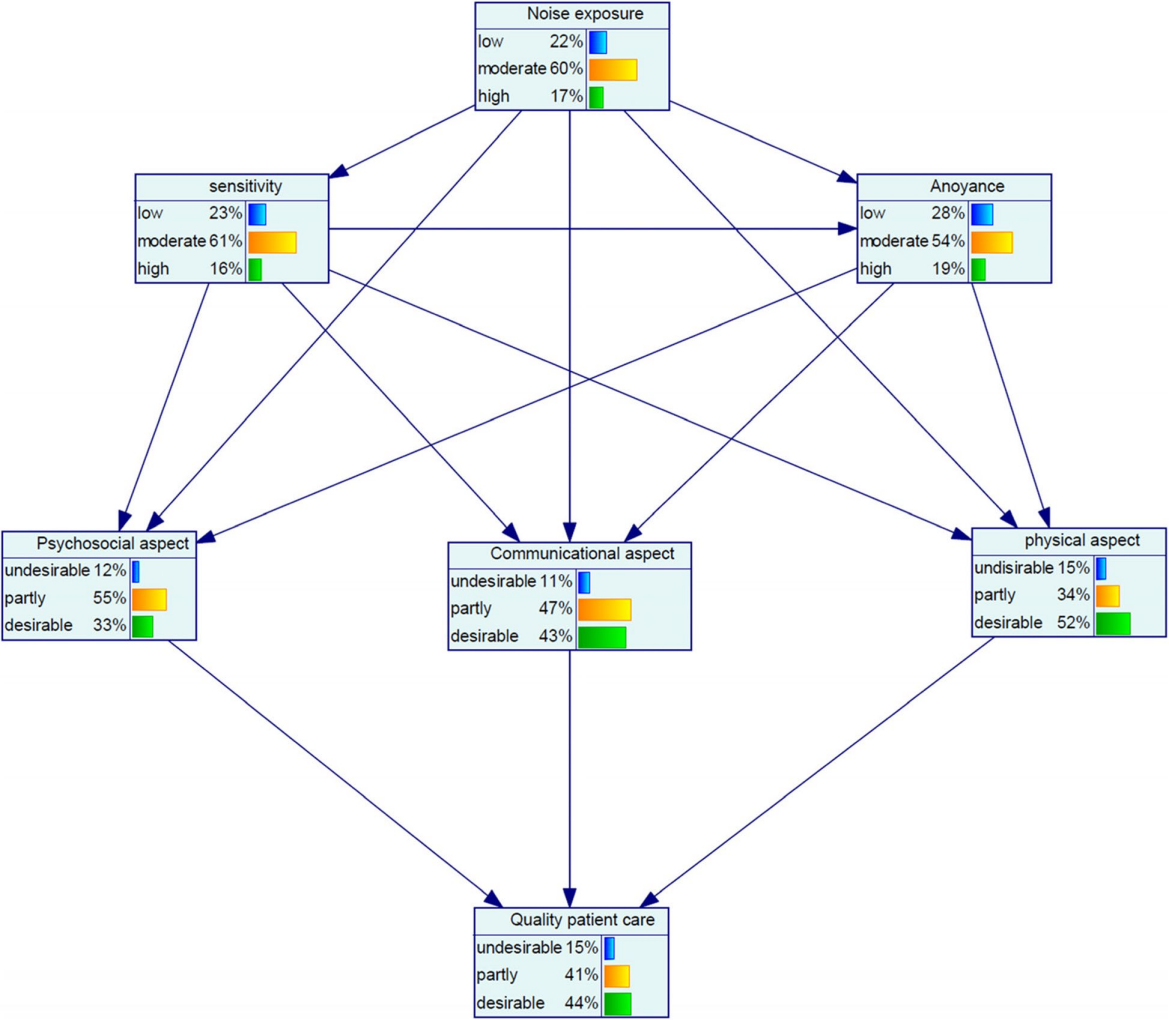
The dependencies among the marginal probabilities of the studied variables based on the Bayesian network model

**Fig. 2:**
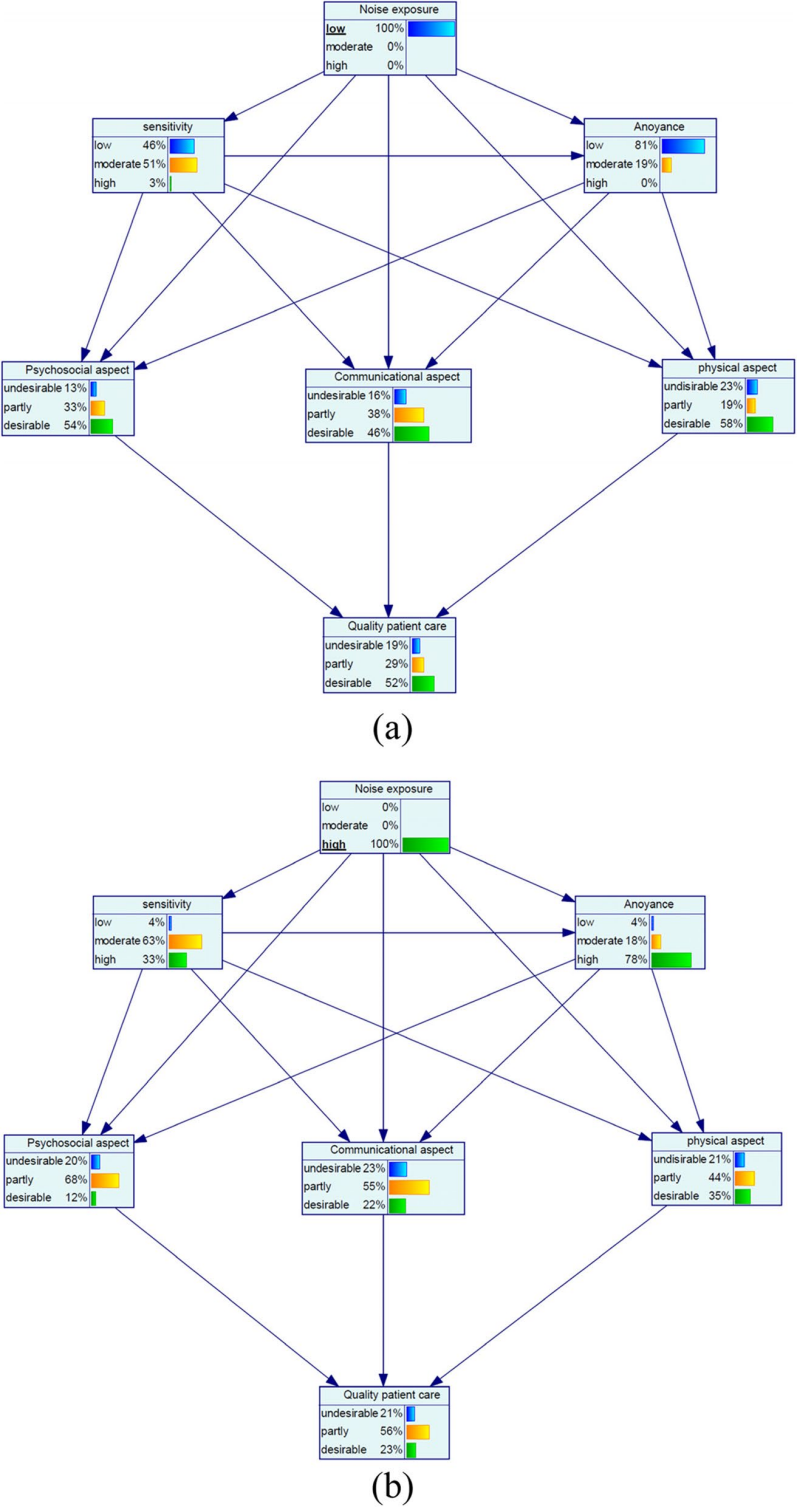
Sensitivity analysis on noise exposure: (**a**) low and (**b**) high

Figure 1 shows the dependencies among the marginal probabilities of the studied variables based on the Bayesian network model. Table 4 also reports the sensitivity analyses for noise parameters. At the low noise exposure with the probability of 100%, the probability of the variables of low sensitivity, low annoyance, and desirable quality of patient care increased by 23, 53, and 8%, respectively (Fig. 2(a)). Moreover, at the high noise exposure with the probability of 100%, the probability of the variables of high sensitivity and high annoyance increased by 17 and 59%, respectively, but desirable quality of patient care decreased by −21% (Minus sign is indicative of a decrease) (Fig. 2(b)). Regarding aspects of quality of patient care for the state of low noise exposure, most increase was related to the desirable psychosocial aspect by an increase of 21%. Moreover, desirable communicational and physical aspects increased by 3 and 6%, respectively (Fig. 2(a)). For the state of high noise exposure, undesirable communicational aspect, undesirable psychosocial aspect and undesirable physical aspect increased by 12, 8 and 6%, respectively (Fig. 2(b)).

**Table 4:** Sensitivity analysis for noise parameters

Parameter	Level	Low (100%)				High (100%)			
		Noise exposure	Annoyance	Sensitivity	Noise exposure and sensitivity	Noise exposure	Annoyance	Sensitivity	Noise exposure and sensitivity
Annoyance	Low	+ 53%	–	+ 40%	+ 72%	- 24%	–	- 22%	- 28%
	Moderate	−35%	–	- 22%	- 54%	- 36%	–	- 13%	- 49%
	High	−19%	–	- 19%	- 19%	+ 59%	–	+ 34%	+ 76%
Sensitivity	Low	+ 23%	–	–	–	- 19%	–	–	–
	Moderate	+ 10%	–	–	–	+ 2%	–	–	–
	High	−13%	–	–	–	+ 17%	–	–	–
Quality of patient care	Undesirable	+ 4%	- 6%	- 4%	- 3%	+ 6%	+ 11%	+ 6%	+ 7%
	Partly desirable	−12%	- 14%	- 9%	- 13%	+ 15%	+ 12%	+ 8%	+ 20%
	Desirable	+ 8%	+ 19%	+ 13%	+ 16%	- 21%	- 23%	- 14%	- 26%
Psychosocial aspect	Undesirable	+ 1%	- 6%	- 3%	- 1%	+ 8%	+ 15%	+ 2%	- 1%
	Partly desirable	−22%	- 15%	- 10%	- 18%	+ 13%	+ 8%	+ 7%	+ 24%
	Desirable	+ 21%	+ 21%	+ 13%	+ 19%	- 21%	- 23%	- 9%	- 22%
Communicational aspect	Undesirable	+ 5%	- 7%	- 5%	- 7%	+ 12%	+ 4%	0%	+ 15%
	Partly desirable	- 9%	- 6%	- 3%	+ 5%	+ 8%	+ 11%	+ 13%	+ 11%
	Desirable	+ 3%	+ 12%	+ 7%	+ 1%	- 21%	- 16%	- 14%	- 27%
Physical aspect	Undesirable	+ 8%	- 8%	- 6%	- 8%	+ 6%	+ 9%	+ 3%	+ 11%
	Partly desirable	−15%	- 12%	- 9%	- 15%	+ 10%	+ 16%	+ 17%	+ 22%
	Desirable	+ 6%	+ 19%	+ 14%	+ 22%	- 17%	- 26%	- 21%	- 33%

**Fig. 3:**
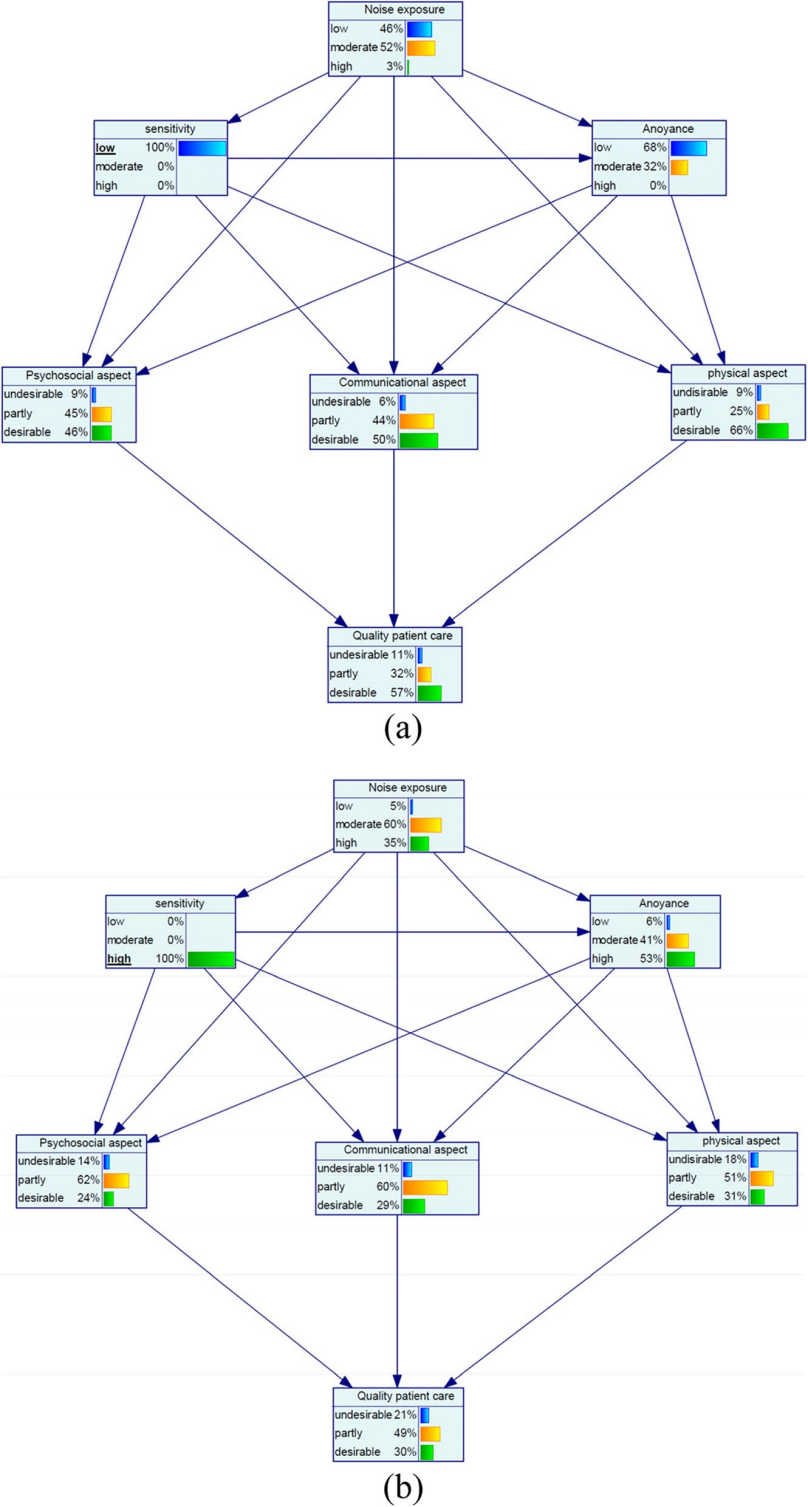
Sensitivity analysis on noise sensitivity: (**a**) low and (**b**) high

For the low noise sensitivity with the probability of 100%, the probability of the variables of the low annoyance and desirable quality of patient care increased by 40 and 13%, respectively (Fig. 3(a)). Concerning the high noise sensitivity with the probability of 100%, high annoyance and desirable quality of patient care increased by 34% and − 14%, respectively (Minus sign indicative of a decrease) (Fig. 3(b)). Among aspects of quality of patient care for the state of low noise sensitivity, most change was related to the desirable physical aspect with a 14% increase and for the state of high noise sensitivity, most change belonged to the desirable physical aspect with a − 21% decrease (Fig. 3(a and b)).

**Fig. 4:**
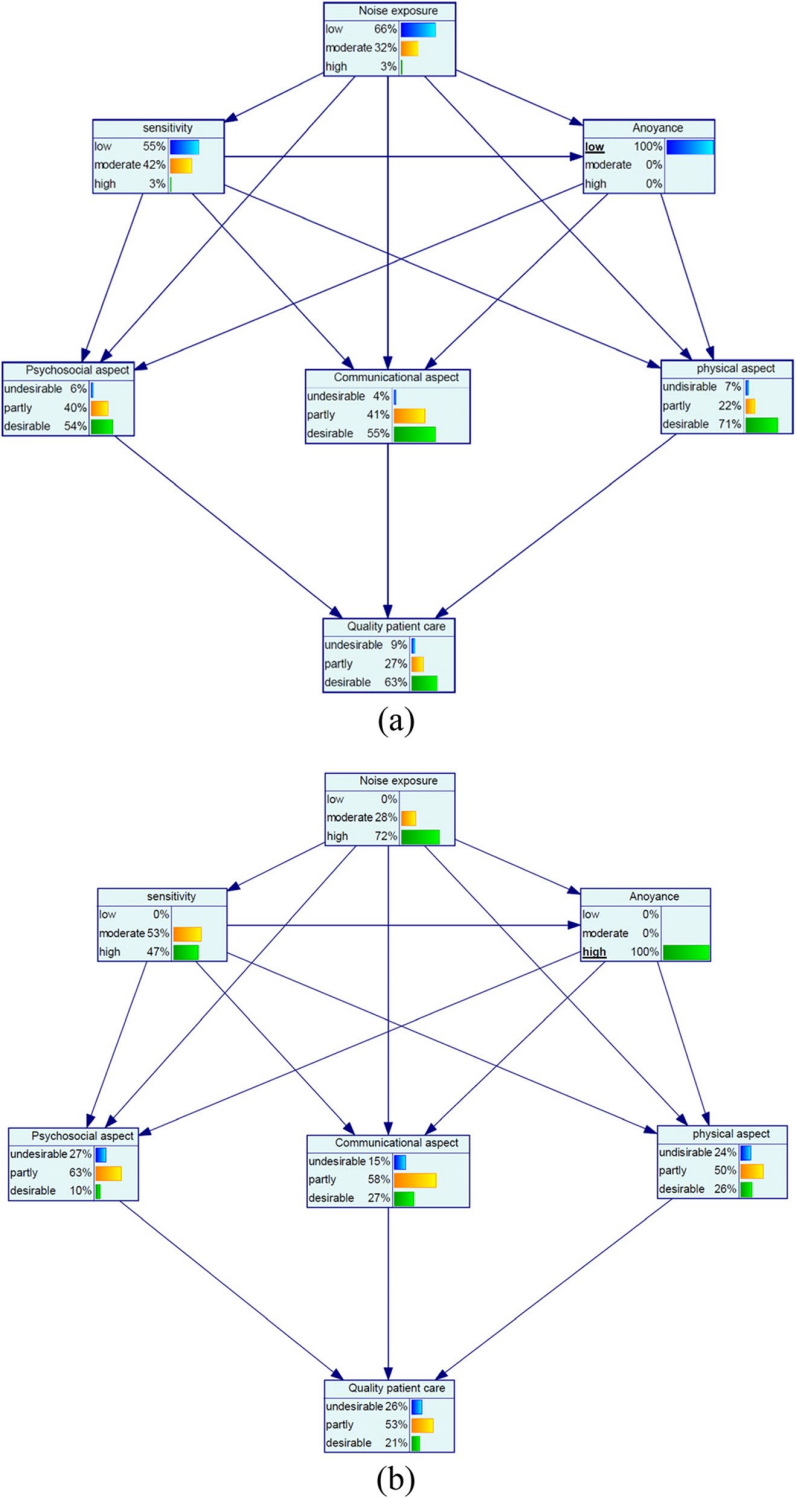
Sensitivity analysis on noise annoyance: (**a**) low and (**b**) high

At the low noise annoyance with the probability of 100%, the probability of the desirable quality of patient care increased by 19% and for the high noise annoyance with the probability of 100%, the probability of the desirable quality of patient care decreased by 23%. Among aspects of quality of patient care for the state of low noise annoyance, most increase was related to the desirable psychosocial aspect with a 21% increase and for the state of high noise annoyance, most increase belonged to the undesirable psychosocial aspect with a 15% increase (Fig. 4(a and b)).

**Fig. 5:**
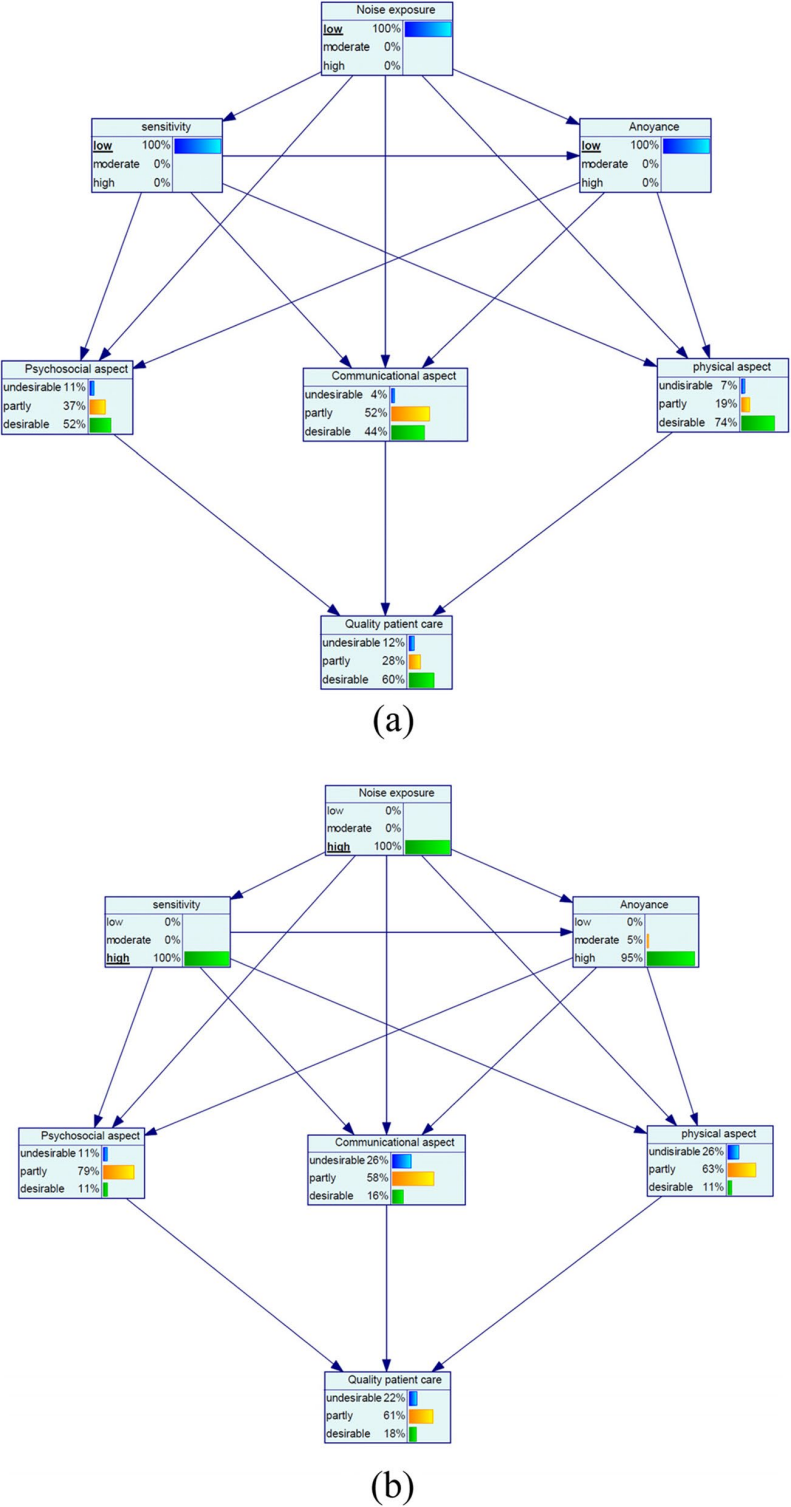
Sensitivity analysis on simultaneous noise exposure and noise sensitivity: (**a**) low and (**b**) high

Furthermore, at the concurrently low noise exposure and low noise sensitivity with the probability of 100%, the desirable quality of patient care increased by 16% and at the concurrently high noise exposure and high noise sensitivity, the desirable quality of patient care decreased by 26%. Among the aspects of quality of patient care for the low noise/low sensitivity states, most increase was related to the desirable physical aspect with a 22% increase and for the high noise/high sensitivity states, most increase belonged to the undesirable communication aspect with a 15% increase (Fig. 5(a and b)).

Table 5 represents the computed effective value for the association among the modeled variables. In this table, the parent (independent) is a variable influencing the child (dependent) and the child is a variable affected by parent. Most influence values belonged to the association of noise exposure and annoyance (0.636), relationship of quality of patient care with physical aspect (0.475), with communicational aspect (0.441), and with psychosocial aspect (0.439), respectively. Annoyance had the highest association with the physical aspect (0.400) and nurses’ noise sensitivity had the greatest association with the communicational aspect (0.283). Also, the association between noise exposure and annoyance (0.636) was greater compared to its association with sensitivity (0.257).

**Table 5:** The calculated influence value of the modeled relationships

Parent	Child	Influence value
Noise exposure	Annoyance	0.636
Physical aspect	Quality of patient care	0.475
Communicational aspect	Quality of patient care	0.441
Psychosocial aspect	Quality of patient care	0.439
Sensitivity	Annoyance	0.429
Annoyance	Physical aspect	0.400
Noise exposure	Psychosocial aspect	0.330
Annoyance	Psychosocial aspect	0.327
Annoyance	Communicational aspect	0.308
Noise exposure	Communicational aspect	0.306
Sensitivity	Communicational aspect	0.283
Noise exposure	Physical aspect	0.282
Sensitivity	Physical aspect	0.275
Noise exposure	Sensitivity	0.257
Sensitivity	Psychosocial aspect	0.245

**Fig. 6:**
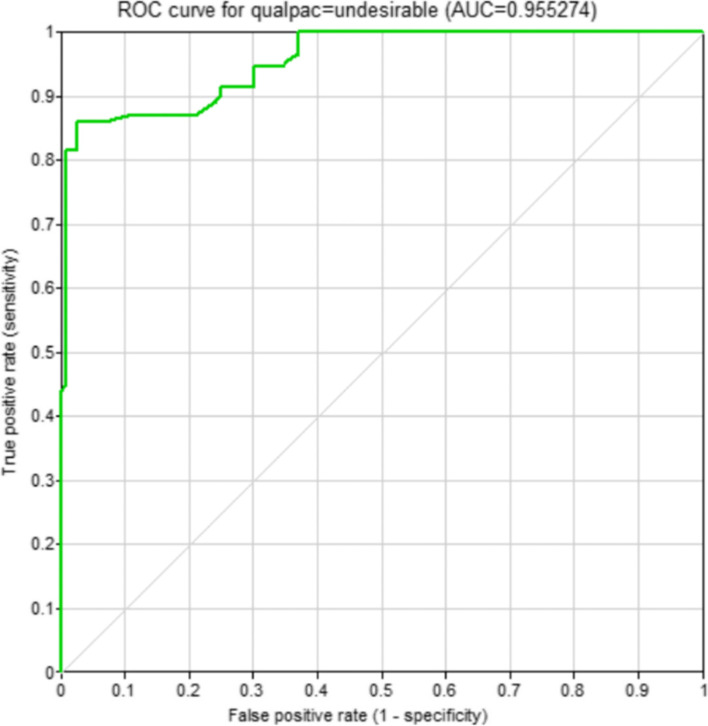
The ROC curve

A ROC curve depicted to examine the validity of the fitted Bayesian model is shown in Fig. 6. The area under the curve was equal to 0.955. As reported in Table 6, the confusion matrix related to the classification of the quality of patient care status was also calculated and presented in Table 6. Moreover, the values of the sensitivity, specificity, and accuracy of the model were computed as 0.860, 0.971, and 0.842, respectively.

**Table 6:** The confusion matrix related to the classification of the quality of patient care status

	Predicted				
	Desirable	Partly desirable	Undesirable		
Sensitivity = 0.860	13	0	80	Undesirable	Actual
Specificity = 0.971	8	11	0	Partly desirable	
Accuracy = 0.842	85	5	7	Desirable	

## Discussion

The World Health Organization has recommended that noise levels in hospital wards should not exceed 30 and 35 dBA during night and day time, respectively [25]. In this study, nurses’ noise exposure was beyond the standard developed by WHO and its mean was 48.80, 54.92, and 61.75 for low, moderate, and high noise exposure groups, respectively. Therefore, it is expected that some harmful effects such as health effects, decreased performance, absenteeism, and decreased cognitive functions occur among nurses even at the lower exposure levels. Regarding that, this study was conducted to predict the contributing role of noise, sensitivity, and annoyance on quality of patient care. Based on the assumed model, noise as an environmental stressor and sensitivity to noise as an individual factor can affect annoyance and in turn, these factors can exert their effect on the quality of care as one of the performance indicators in nursing occupations.

At the low noise exposure with the probability of 100% and at the low noise sensitivity with the probability of 100%, the probability of the variables of low annoyance increased by 53, and 40%, respectively (Fig. 2(a)). Moreover, at the concurrently low noise exposure and low noise sensitivity with the probability of 100%, the low annoyance increased by 72%. This shows that annoyance is more influenced by noise exposure compared to sensitivity. These results are consistent with Monazzam et al. study, where the effect size of noise and sensitivity on annoyance were 0.57 and 0.52, respectively [26]. Moreover other studies have concluded that annoyance perceived by nurses is one of the main harmful effects of exposure to hospital noise [9, 27]. Noise annoyance is caused due to exposure to noise and also the level of individual sensitivity to the noise. In other words, noise sensitivity is considered as a mediator between the noise exposure and annoyance. Noise-sensitive people are more vulnerable to the negative health effects induced from noise exposure. As a result, it is reasonable to predict that they found noise more annoying [28].

Concerning the quality of patient care, it can be seen that at the low noise exposure, low annoyance, and low sensitivity with the probability of 100%, the outcome of interest increased by + 8%, + 19%, and + 13%, respectively. Moreover, at the concurrently low noise exposure and low noise sensitivity with the probability of 100%, the desirable quality of patient care increased by 16%. This shows that the quality of patient care is more affected by annoyance than noise exposure and sensitivity. This reasonable result is expected because annoyance is regarded as the outcome of both noise exposure and sensitivity. Regarding the effect of noise exposure on quality of patient care, Terzi et al. confirmed that noise adversely affected the nurse’s satisfaction and anxiety and these unwanted outcomes can threaten the quality of patient care [29]. In this study, the quality of patient care is measured by three dimensions including psychosocial, communicational, and physical aspects. The results indicated that reduced noise could increase the desirable aspects of the aforementioned dimensions by 21%, 6%, and 3%, respectively. There are robust reports that noise can impair cognitive performance and productivity, disturb communication, and compromise speech privacy [30, 31]. Moreover, noise can influence the psychosocial aspect of health, annoyance, psychosocial stress, and behaviors [32, 33]. Disturbed communication can be followed by misunderstanding of the patient and colleagues’ needs and in turn, reduce the quality of patient care. Moreover, it is well documented that annoyance as a negative psychological reaction induced by noise exposure can trigger more adverse health effects. Annoyance as a mediator can exert harmful effects of noise exposure on stress responses and other health consequences [34]. Stress responses also can impair functional and behavioral stability of nurses and in turn, influence their performance and quality of care. Moreover, Abbasi et al. stated that noise annoyance had a significant adverse impact on job stress and blood pressure and it decreased job satisfaction [15, 35, 36]. These consequences can justify the effect of noise and noise annoyance on the quality of patient care. In other words, noise and noise annoyance can lead to decreased quality of patient care through these consequences. In a study on the effect of ambient noise on annoyance and potential health effects, Amoaty et al. reported that medical staff complained about noise and its effect on overall work performance and loss of concentration [37]. Therefore, it can be expected that noise can affect quality of care through these adverse effects.

The results of our study showed that reduced sensitivity to noise decreases high levels of annoyance by 19%. Moreover, desirable quality of patient care, psychosocial, communicational, and physical aspects increased by + 13%, + 13%, + 7%, and + 14%, respectively. In addition to noise, it is well known that noise sensitivity can predict noise annoyance [26, 38]. Therefore, similar to the noise, it can be stated that noise sensitivity exerted its effect on the quality of patient care through annoyance. According to the research literature, noise sensitivity can have negative psychological and physiological effects. Abbasi et al. stated that noise sensitivity has a negative impact on job stress and it can act in a synergistic manner with noise exposure on job stress [36]. Khan et al. reported that as the noise sensitivity raised by 1 unit, the cortisol level raised by 0.032 μg/dL [39]. Cortisol secretion as the main cause of increased stress has a significant adverse effect on individual performance [40]. Thus, it is expected that noise-sensitive nurses release more stress hormones and in turn, their performance and quality of patient care will be reduced. In addition to the subjective and psychological effects, noise sensitivity can trigger some physiological responses such as cardiovascular disease, hypertension and immune response [38, 41–43]. As a result, all of these reactions that may occur in noise-sensitive nurses may have an unfavorable influence on patient care quality. Furthermore, it has been noted that noise-sensitive people are more vulnerable to other environmental stressors and they may become inefficient and disrupted while they are exposed to stressors such as noise. According to the findings of the current study, when noise-sensitive nurses are exposed to noise, they perceive noise more annoying and in turn their quality of patient care will be further declined.

Alongside the valuable results obtained in this study, there are several limitations that future researchers will want to address. As a limitation, the noise level was the only studied variable and the effect of noise frequency and psychoacoustics indices were not considered. Moreover, the study was conducted during the COVID-19 pandemic. This issue can affect both noise pollution levels in hospitals and the quality of patient care regardless of the noise impacts. This study was cross-sectional and therefore limitations such as lacking temporal relationship and causal investigation are to be expected. To overcome these limitations, conducting longitudinal studies should be considered for future research. Moreover, using self-report instruments to collect individual data may cause some biases that should be covered off somewhat by applying objective data gathering methods.

## Conclusion

The results of this study concluded that hospital noise and noise sensitivity as environmental stressors and individual traits can interact with each other and in turn influence nurses’ perception of noise and quality of patient care. Annoyance as a subjective reaction to the noise and personal noise sensitivity has a significant adverse impact on the quality of patient care. In addition to this, noise and sensitivity had a separate direct adverse effect upon the quality of patient care and their co-occurrence potentially reduced the quality of patient care.

## Data Availability

The datasets used and analyzed during the current study are available from the corresponding author on reasonable request.
